# Patient-ventilator asynchronies during mechanical ventilation: current knowledge and research priorities

**DOI:** 10.1186/s40635-019-0234-5

**Published:** 2019-07-25

**Authors:** Candelaria de Haro, Ana Ochagavia, Josefina López-Aguilar, Sol Fernandez-Gonzalo, Guillem Navarra-Ventura, Rudys Magrans, Jaume Montanyà, Lluís Blanch, the Asynchronies in the Intensive Care Unit (ASYNICU) Group, Candelaria de Haro, Josefina López-Aguilar, Rudys Magrans, Sol Fernández-Gonzalo, Gemma Gomà, Encarna Chacón, Ana Ochagavia, Lluís Blanch, Jaume Montanya, Bernat Sales, Enrico Lena, Umberto Lucangelo, Rafael Fernández, Carles Subirà, Guillermo M. Albaiceta, Gastón Murias, Robert M. Kacmarek

**Affiliations:** 1 Critical Care Center, Hospital Universitari Parc Taulí, Institut d’Investigació i Innovació Parc Taulí I3PT, Universitat Autònoma de Barcelona, Parc Taulí 1, 08208 Sabadell, Spain; 2 CIBERES, Instituto de Salud Carlos III, Madrid, Spain; 3 CIBERSAM, Instituto de Salud Carlos III, Madrid, Spain; 4 Better Care, Barcelona, Spain

**Keywords:** Patient-ventilator interaction, Asynchronies, Mechanical ventilation, Outcome, Heart lung interaction, Psychological disorders, Cognitive, ICU, Critically ill, Big data

## Abstract

**Background:**

Mechanical ventilation is common in critically ill patients. This life-saving treatment can cause complications and is also associated with long-term sequelae. Patient-ventilator asynchronies are frequent but underdiagnosed, and they have been associated with worse outcomes.

**Main body:**

Asynchronies occur when ventilator assistance does not match the patient’s demand. Ventilatory overassistance or underassistance translates to different types of asynchronies with different effects on patients. Underassistance can result in an excessive load on respiratory muscles, air hunger, or lung injury due to excessive tidal volumes. Overassistance can result in lower patient inspiratory drive and can lead to reverse triggering, which can also worsen lung injury. Identifying the type of asynchrony and its causes is crucial for effective treatment.

Mechanical ventilation and asynchronies can affect hemodynamics. An increase in intrathoracic pressure during ventilation modifies ventricular preload and afterload of ventricles, thereby affecting cardiac output and hemodynamic status. Ineffective efforts can decrease intrathoracic pressure, but double cycling can increase it. Thus, asynchronies can lower the predictive accuracy of some hemodynamic parameters of fluid responsiveness.

New research is also exploring the psychological effects of asynchronies. Anxiety and depression are common in survivors of critical illness long after discharge. Patients on mechanical ventilation feel anxiety, fear, agony, and insecurity, which can worsen in the presence of asynchronies. Asynchronies have been associated with worse overall prognosis, but the direct causal relation between poor patient-ventilator interaction and worse outcomes has yet to be clearly demonstrated.

Critical care patients generate huge volumes of data that are vastly underexploited. New monitoring systems can analyze waveforms together with other inputs, helping us to detect, analyze, and even predict asynchronies. Big data approaches promise to help us understand asynchronies better and improve their diagnosis and management.

**Conclusions:**

Although our understanding of asynchronies has increased in recent years, many questions remain to be answered. Evolving concepts in asynchronies, lung crosstalk with other organs, and the difficulties of data management make more efforts necessary in this field.

## Background

**Fig. 1:**
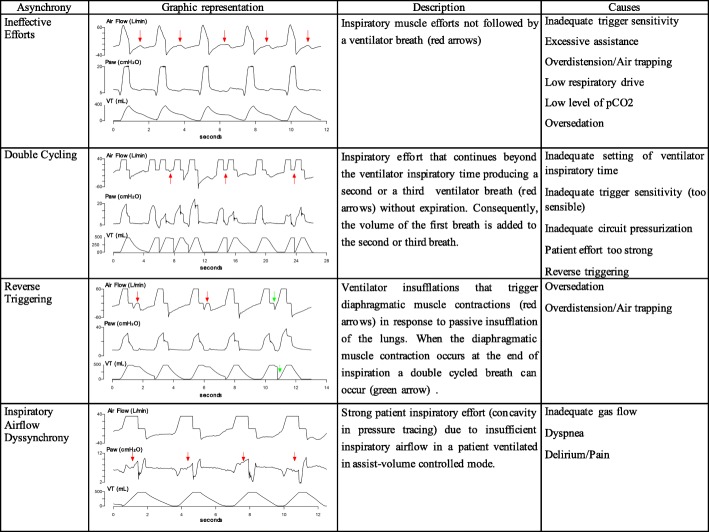
Representation and description of the most common asynchronies. Ineffective efforts, double cycling, reverse triggering, and inspiratory airflow dyssynchrony are graphically represented and described together with their causes. Red arrows indicate where the asynchrony described is present

**Fig. 2:**
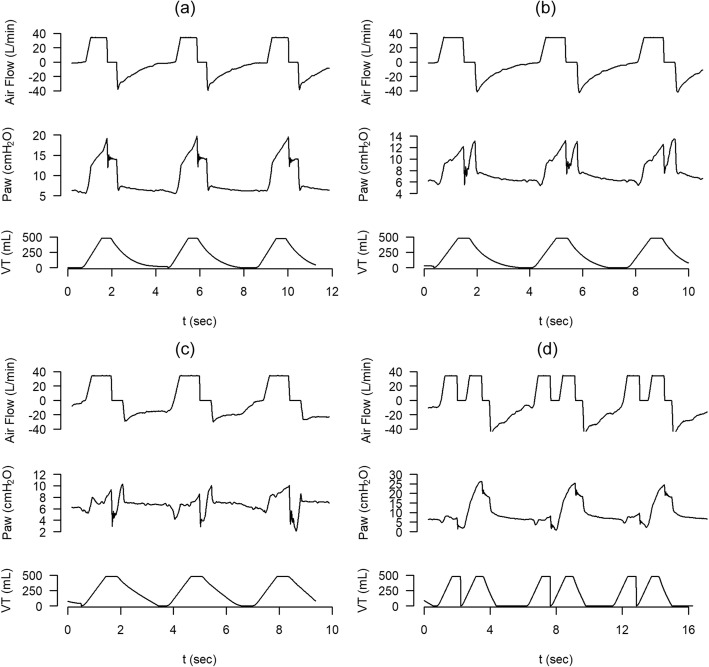
Inspiratory airflow dyssynchrony. Sequence of airflow and airway pressure waveforms corresponding to a same patient in the same day ventilated in assist volume control mode. Set airflow is insufficient for the patient’s needs and originated different degrees of airflow dyssynchrony or starvation. **a** Mild airflow dyssynchrony. **b**, **c** The progression of airflow dyssynchrony through a more severe stage. **d** The appearance of double cycling secondary to a huge and large inspiratory effort

Invasive mechanical ventilation is the most common means of life support applied in critical care medicine. Although mechanical ventilation often helps save lives, the mortality associated with this technique is very high. In addition, survivors of mechanical ventilation may experience significant long-term morbidity resulting in substantially reduced functional status and ability to complete activities of daily living [1–3]. Optimal patient-ventilator interaction is crucial to assure comfort with mechanical ventilation and to avoid poor outcomes [4]. Patient-ventilator asynchronies (PVA) are the consequence of a mismatch between patients’ needs and the assistance delivered by the ventilator. PVA can be classified depending on the phase of the respiratory cycle in which they occur. The most frequent PVA are ineffective efforts, followed by double cycling. Its causes, consequences, and management vary depending on the type (Figs. 1 and 2) [5, 6]. This review article aims to summarize what is known about patient-ventilator interaction and asynchronies in mechanical ventilation, to show its effects on outcomes, and to describe new directions in research about these questions.

## Main text

### Evolving concepts on patient-ventilator interaction and asynchronies

Patient-ventilator interaction has been investigated for years [5, 7, 8]. Authors have examined various factors related to ventilator mode, ventilator settings, and patient characteristics that can affect patient-ventilator interaction [4, 5, 9], and have identified many types of asynchronies. Some factors associated with different types of asynchronies have been analyzed, and different mechanisms to reduce their incidence have been studied [10]. Nevertheless, detecting PVA remains a challenge, requiring the application of advanced knowledge about respiratory physiology to interpret ventilator waveforms by analyzing their shape during different periods of the breath cycle (inspiration, transition from inspiration to expiration, and expiration) [5, 6, 8, 11, 12]. Until recently, such analyses required the physical presence of an expert physician at the bedside and were thus only possible during brief, intermittent periods.

PVA occurs when there is a mismatch between the ventilator and the patient in terms of demand or breath delivery timing. Recently, Pham et al. [6] proposed a classification of PVA based on the appropriateness of the level of assistance provided by the ventilator. Assistance is deemed insufficient when the ventilator fails to meet the patient’s flow demand. Inspiratory airflow dyssynchrony due to insufficient ventilator airflow (also named flow starvation) results in the patient’s inspiratory effort continuing beyond the ventilator’s inspiratory time. When the patient’s effort is strong enough, a second breath can be triggered with no or minimal expiration (called double cycling or breath stacking), resulting in a potentially dangerous increase in tidal volume. Inspiratory airflow dyssynchrony develops mainly when ventilators are set to deliver fixed flow and/or lower tidal volumes in patients with high inspiratory flow demands that vary from breath to breath [13, 14]. Potential consequences of low assistance are excessive load on the respiratory muscles, air hunger promoting limbic, paralimbic, and cerebellar activation in the brain [15], and ventilator-induced lung injury due to excessive tidal volume. Moreover, strong inspiratory efforts can increase transvascular pressure gradients and tidal recruitment associated with pendelluft flow and regional lung overdistension [16, 17]. Vigorous spontaneous efforts impact non-dependent and dependent lung regions differently, increasing inspiratory distension but also apparently worsening injury in the dependent lung because diaphragm contraction is poorly transmitted to the remainder of the pleural surface and is thus “confined” to the dependent lung [18]. It can be difficult to detect asynchronies due to insufficient assistance.

By contrast, assistance is deemed excessive when the ventilator provides flow in excess of the patient’s demand. Patients with low inspiratory drive due to sedation or excessive ventilator assistance can develop ineffective efforts occurring during either inspiration or expiration, delayed or prolonged cycling, and reverse triggering [6]. The concept of reverse triggering is evolving. Reverse triggering is a frequently under-recognized form of PVA in which the patient’s respiratory center is activated in response to a passive insufflation of the lungs. This PVA originates in respiratory muscle contractions triggered by the ventilator [19]. The physiologic mechanism responsible for reverse triggering seems to be related with mechanoreceptors in the muscles and/or chest wall or in complex spinal reflexes [6]. Passive insufflation of the lungs activates the patient’s neurological respiratory center [20]. Recent research has found that reverse triggering could occur not only in patients with acute respiratory distress syndrome or diagnosed of brain death but in all patients receiving mechanical ventilation. Since reverse triggering might be more frequent than expected and could be associated with lung or diaphragm injury, the incidence and causes of reverse triggering warrant urgent investigation [6, 21, 22]. Esophageal pressure monitoring can help to identify this PVA in deeply sedated patients: a drop in esophageal pressure can be related to diaphragmatic contractions triggered by ventilator insufflations [19]. Reverse triggering can result in stretching in the dependent lung. Proportional to negative intrathoracic pressure, stretching due to reverse triggering can be equivalent to that caused by applying 15 ml/kg tidal volume. Yoshida et al. [18] recently demonstrated how reverse triggering can worsen pre-existing lung injury through a pendelluft effect from non-dependent lung areas toward dependent areas due to poor transmission of the diaphragm contraction across the pleural surface in an injured lung. Moreover, reverse triggering can result in increased strain and stretch due to breath stacking during double cycling caused by insufficient assistance [19, 23].

In a recent publication, we used the term double cycling to refer to both reverse-triggered or patient-triggered mechanical breaths occurring at any point in mechanical ventilation [21]. Other authors [24] use double cycling only when the first breath in a reverse-triggering event is a ventilator-programmed breath not triggered by the patient. Only the breaths originated by a patient’s high inspiratory drive were considered as double triggering. Moreover, reverse triggering that does not cause double cycling can be considered an ineffective effort during the different phases of inspiration. Several authors have speculated that reverse triggering without double cycling can cause lengthening contractions of the diaphragm and with double cycling breath-stacking with increased tidal volume in assist pressure control mode, and both tidal volume and airway pressure in assist volume control mode. Recent investigations suggest that reverse triggering is common in critical patients. Interestingly, de Haro et al. [21] found that one third of double-cycling breaths were reverse-triggered. Clinicians must differentiate between double cycling due to insufficient assistance and double cycling due to reverse triggering because these phenomena call for different treatments. Double cycling due to insufficient assistance is associated with rapid respiratory rates, low ventilator airflow, and short ventilator inspiratory time [21, 25]; by contrast, double cycling due to reverse triggering is associated with deep sedation in patients not triggering the ventilator. However, the mechanisms involved in reverse triggering are poorly understood, so the best treatment approach remains to be determined [6]. At present, detecting these types of PVA requires trained observers analyzing waveforms on ventilator screens at the bedside.

### Heart-lung interaction in patients with asynchronies

The heart and lungs are anatomically and functionally linked. The interactions between cardiovascular and respiratory physiology are very complex and include effects related to changes in intrathoracic pressure and lung volumes [26, 27]. Moreover, the hemodynamic effects of ventilation and asynchronies depend on the hemodynamics’ stability and the previous status of the cardiopulmonary system.

In mechanically ventilated patients, the inspiratory increase in intrathoracic pressure reduces venous return by increasing right atrial pressure and reduces left ventricular afterload by decreasing transmural left ventricular systolic pressure. Conversely, it can also increase the afterload of the right ventricle considerably. Increased right ventricular afterload results from progressive increases in transpulmonary pressure (difference between alveolar pressure and pleural pressure) associated with increasing lung volume; this effect can be especially important in patients with acute respiratory distress syndrome. Moreover, left ventricular preload can be affected by changes in right ventricular preload and by ventricular interdependence [26–28]. Interestingly, heart-lung interactions may be useful to assess fluid responsiveness in critical care [28, 29].

urthermore, changes in heart load conditions can in turn lead to lung injury. In a recent experimental study, Katira et al. [30] showed that abrupt deflation after sustained inflation can cause acute lung injury; in critical patients, deflation could occur when positive end-expiratory pressure is removed or the patient is disconnected from the ventilator. Apparently, lung injury results from acute left ventricular decompensation (increased left ventricular preload and afterload), which raises the pressure in the pulmonary microvasculature, injuring the endothelium and causing edema, which is in turn potentiated by the surge in pulmonary perfusion. Whether this phenomenon observed in experimental animals could occur in patients under mechanical ventilation warrants further investigation.

Unfortunately, the hemodynamic effects caused by different PVA have not been extensively studied. In theory, ineffective efforts decrease intrathoracic pressure and could consequently increase venous return and right ventricular filling. However, there are no physiological data to confirm this hypothesis. On the other hand, our group recently showed that the tidal volume accumulated during double cycling is very high, sometimes even doubling that of normal breaths in volume-targeted modes [21]. In addition, the peak pressure of the second breath is generally greater than that of the first. Both increased volume and pressure could significantly affect preload and afterload, but the hemodynamic consequences of these effects have not been evaluated.

Although dynamic parameters based on heart-lung interactions such as pulse pressure variation (PPV) and stroke volume variation (SVV) accurately predict fluid responsiveness in patients passively adapted to the ventilator, these parameters are not good predictors of fluid responsiveness in patients with spontaneous respiratory activity probably due to multiple causes such as increased preload induced by negative intrathoracic pressure during patient inspiration and the variability of the breathing pattern [31]. In the presence of asynchronies, this effect could be magnified.

In a recent study examining whether PVA affected PPV’s ability to predict fluid responsiveness in patients receiving pressure support ventilation, Messina et al. [32] compared 27 patients without PVA versus 27 with PVA as determined by visual inspection of ventilator waveforms. The area under the receiver operating characteristic curve was 0.86 (CI 0.68–0.96) in patients without PVA but only 0.53 (CI 0.33–0.73) in those with PVA (*p* = 0.018); PPV ≥ 13% predicted fluid responsiveness with 78% sensitivity and 89% specificity in patients without PVA but only 36% sensitivity and 46% specificity in those with asynchronies. PVA significantly affected PPV prediction of fluid responsiveness (OR 8.8 [2.0–38.0]; *p* = 0.003). They hypothesize that PVA affect the cyclical changes in intrathoracic pressure, resulting in unpredictable and persistent variations of right ventricular preload and left ventricular stroke volume, thereby altering the reliability of PPV in assessing fluid responsiveness. Nevertheless, more physiological and clinical data are needed to determine the implications of these findings in clinical practice.

### Can asynchronies impact major outcomes?

PVA are frequent but underdiagnosed, and they have been associated with worse prognosis: discomfort; sleep disorders [33], which increase the need for sedatives [34]; prolongation of mechanical ventilation [7, 35]; increased intensive care unit (ICU) and hospital stays [33]; and increased mortality [4]. Thus, it seems crucial to take action to reduce their incidence [5]. Nevertheless, a direct causal relation between poor patient-ventilator interaction and worse outcomes has yet to be clearly demonstrated, and there is no direct evidence to demonstrate that reducing PVA guarantees better outcomes.

To clarify whether PVA are a direct causative factor of worse outcomes, it is necessary to identify and quantify the occurrence of PVA throughout the entire period of mechanical ventilation. To this end, monitoring systems have recently been developed to enable such analyses, and these systems are helping elucidate the potential harmful physiological effects of different types of PVA [36, 37].

Monitoring systems have made possible to analyze the magnitude of PVA and how they are distributed over time, which are crucial factors in the evaluation of the impact of PVA on clinical outcomes. In a secondary analysis, Blanch et al. [4] found that although patients with an asynchrony index > 10% had similar rates of reintubation and tracheostomy compared to those with lower rates, an asynchrony index > 10% was associated with higher ICU and hospital mortality and with a trend toward longer duration of mechanical ventilation. Beyond the frequency of PVA, Vaporidi et al. [38] focused on the presence of clusters of ineffective efforts as well as their power and duration, finding that all these aspects were associated with prolonged mechanical ventilation and higher hospital mortality and highlighting the need to examine different dimensions of patient-ventilator interaction. Finally, Rue et al. [39] used Bayesian joint modeling of bivariate and competing risks data to investigate the added value of adding information about the rate of PVA to Sequential Organ Failure Assessment (SOFA) scores to predict outcomes. They found an association between the asynchrony index and live discharge, but including this information did not improve the accuracy of the prognosis of the SOFA score alone. They concluded that a more detailed analysis of PVA, together with other multidimensional data, would be necessary to confirm a causal role on ventilated patients’ outcomes and comorbidities. It also remains to be demonstrated whether strategies to optimize patient-ventilator interactions improve outcomes.

De Haro et al. [21] analyzed the incidence, mechanisms, and physiologic implications of double cycling in 67 adults continuously monitored while undergoing various modes of volume- and/or pressure-targeted mechanical ventilation for more than 24 h. They found that, as previously observed by others, the volume of stacked breaths resulting from inspiratory airflow dyssynchrony can double the set tidal volume in volume-controlled ventilation [21]. This higher-than-expected tidal volume exceeds the optimal value set for protective ventilation and could harm lung tissue and respiratory muscles [21, 25], thus contributing to ventilation-associated lung injury [21, 24, 25, 40, 41].

Regarding neuropsychological outcomes, new research is exploring how mechanical ventilation is linked to psychological disorders observed in critically ill patients [42, 43]. Anxiety is one of the most common psychological symptoms reported by critically ill patients [44], affecting between 30 and 80% of all patients [45]. Patients on mechanical ventilation report worries about breathlessness, choking, or being left alone [46], and up to 47% of ICU survivors report having felt anxiety and/or fear during mechanical ventilation [47]. It seems that, even after tracheotomy, levels of anxiety do not decrease [44]. However, the direct link between anxiety and asynchronies in mechanically ventilated patients has not been explored yet. Nevertheless, respiratory difficulties, including synchronizing with the respirator, cough, and dyspnea [45, 46], are considered potent drivers of anxiety, agony, and insecurity. Anxiety has been independently associated with dyspnea in critically ill patients undergoing mechanical ventilation, and when ventilator settings are adjusted, dyspnea is reduced in at least a third of patients [48]. Therefore, asynchronies and anxiety in ICU patients could be, somehow, potentially related. Anxiety is a state of psychological distress and physiological discomfort that, if prolonged, delays healing and predisposes to difficulties in weaning from mechanical ventilation [44]. Jubran et al. [49] also found that mechanically ventilated patients with depressive symptoms were three times more likely to experience weaning failure and death. Furthermore, high levels of anxiety often prompt professionals to apply higher sedation doses or restraints, leading to immobility, decreased level of consciousness, and loss of protective reflexes [46].

Despite the importance of the early detection of adverse psychological outcomes during ICU stay, neither anxiety nor depressive symptoms are routinely assessed in mechanically ventilated critically ill patients. In fact, most of the little information available about psychological disorders derives from the studies in ICU survivors [46]. After ICU discharge, 23 to 50% of survivors have generalized, nonspecific anxiety [2, 50, 51], and although it improves over time, anxiety levels in ICU survivors are higher than those observed in medical inpatients (5 to 20%) [2]. Nevertheless, 15 to 43% of survivors continue to have symptoms of anxiety 6 months [51] and 1 year [52] after discharge, and 60% also have other mental health problems such as post-traumatic stress disorder (PTSD) [53–55] and depression [50, 56]. These long-term mental health problems in ICU survivors are often associated with worse quality of life [2, 3].

### Big data techniques applied to large observational databases to improve the management of ventilated patients

In medical research, pragmatic research attempts to approach problems from a broad and, in a sense, a realistic perspective. For example, observational studies of medical interventions may more closely reflect daily clinical practice [57]. However, the main drawback of observational studies is the potential bias and confounding factors, being difficult to establish an independent association between methods/strategies/treatments and outcome variables. The access to large databases of heterogeneous populations with high levels of complexity is key for observational studies to the extent researchers are aware of confounding and able to measure them.

Mechanically ventilated critically ill patients continuously generate huge volumes of data of varying complexity and temporal resolution [36, 58]. Some data (e.g., physiologic waveforms) are generated at very high temporal resolutions, while others are generated at much lower temporal resolutions. Whereas data about laboratory test results might be generated on a daily basis, two medical devices (e.g., multiparameter monitor and ventilator) connected to a mechanically ventilated patient record about 10 different waveforms (electrocardiographic, plethysmographic, capnographic, respiratory, arterial blood pressure, airway pressure, gas flow, volume) at 200 points per second or more, thus producing a total of 172.8 million data points each day or 1.04 billion over the average duration of mechanical ventilation. Traditionally, most of these data are underexploited, becoming unavailable immediately or within 24 to 48 h [58, 59]. Thus, the potential to discover new patterns and extract valuable information to support diagnosis or to predict the time course of a patient’s condition is lost.

During mechanical ventilation, patient-ventilator interaction alternates between periods of complete synchrony and periods with clusters of frequent asynchronies [38]. Yet, physicians optimize mechanical ventilation by assessing waveforms on bedside monitors based on their understanding of the physiological principles involved and evidence from previous studies; however, today’s guidelines for ICUs derive from a scant evidence base, considering the potential evidence base given the massive data generated in the ICU [59]. It should come as no surprise that most physicians perform poorly at managing patient-ventilator interactions and do not recognize common forms of patient-ventilator asynchronies [11], but an equally important problem is that even the most highly skilled professionals can observe only a small proportion of these waveforms, thus increasing the probability of misinterpretation due to sampling errors.

For this reason, there is an urgent need for technological and analytic tools to deal with these pragmatic observational data. Big data promises to help refine our approach to PVA, improving our understanding of the various phenomena, their detection, and their treatment. At present, the continuous and automatic detection of asynchronies is an emerging technological area. Table 1 shows a comparison of some automated methods for patient-ventilator asynchrony detection [60]. However, it can be challenging to implement big data solutions in ICUs. These solutions involve new ethical issues; require investments in technical deployment to resolve problems related to interoperability, network connections, digital storage, etc.; depend on active collaboration among experts from a wide range of areas (physicians, biologists, statisticians, and engineers); and must meet quality standards [61]. Big data solutions to support daily clinical decision making and improve patient care are based on storing and exploring extremely large observational datasets [58, 61–63].

**Table 1:** Comparison of some automated methods for patient-ventilator asynchrony detection

	Type of PVA	Algorithm	Performance
Gholami et al. (2018) [69]	Cycling asynchrony (premature and delayed cycling)	ML: Random forest and *k*-fold cross validation Pressure and airflow signals *N* = 11 patients (1377 breaths)	Se 89–97%, Sp 93–99%, Kappa index 0.9
ventMAP platform Adams et al. (2017) [70]	Double-trigger and breath stacking	Rule-based algorithm Pressure and airflow signals Derivation cohort, *N* = 16 patients (5075 breaths); validation cohort, *N* = 17 patients (4644 breaths)	Se 94–96.7%, Sp 92–98%, Acc 92.2–97.7% (on the validation cohort)
NeuroSync index Sinderby et al. (2013) [71]	Patient-ventilator interaction classification (asynchronous, dyssynchronous or synchronous)	Rule-based timings algorithm EAdi and pressure signals *N* = 24 patients	ICC 0.95 vs. Colombo et al. (2011) [5]
Better Care® system Blanch et al. (2012) [37]	Ineffective efforts during expiration	Rule-based combining digital signal processing techniques and ROC curves Airflow signal Cohort 1: *N* = 8 patients (1024 breaths) Cohort 2: *N* = 8 patients (9600 breaths) with EAdi signal as reference	Se 91.5%, Sp 91.7%, PPV 80.3%, NPV 96.7%, Kappa index 0.797 (vs. the expert’s classification) Se 65.2%, Sp 99.3%, PPV 90.8%, NPV 96.5%, Kappa index 0.739 (vs. EAdi signal)
Gutierrez et al. (2011) [72]	Index for asynchronous/no asynchronous breaths	Time-frequency analysis Airflow signals *N* = 110 patients	Se 83%, Sp 83% when index < 43% for AI > 10%
Mulqueeny et al. (2007) [73]	Ineffective triggering and double triggering	Rule-based and digital signal processing methods Airflow and pressure signals *N* = 20 patients (3343 breaths)	Se 91%, Sp 97%
PVI monitor Younes et al. (2007) [74]	Ineffective efforts	Rule-based Equation of motion from pressure, airflow, and Peso signals *N* = 21 patients	Se 79.7%

Abbreviations: *ML* machine learning, *Se* sensitivity, *Sp* specificity, *ICC* intraclass correlation coefficient, *Acc* overall accuracy, *Peso* esophageal pressure, *PPV* positive predictive value, *NPV* negative predictive value, *ROC* receiver operating characteristics, *AI* asynchrony index according to the definition from Thille et al. [7]

**Fig. 3:**
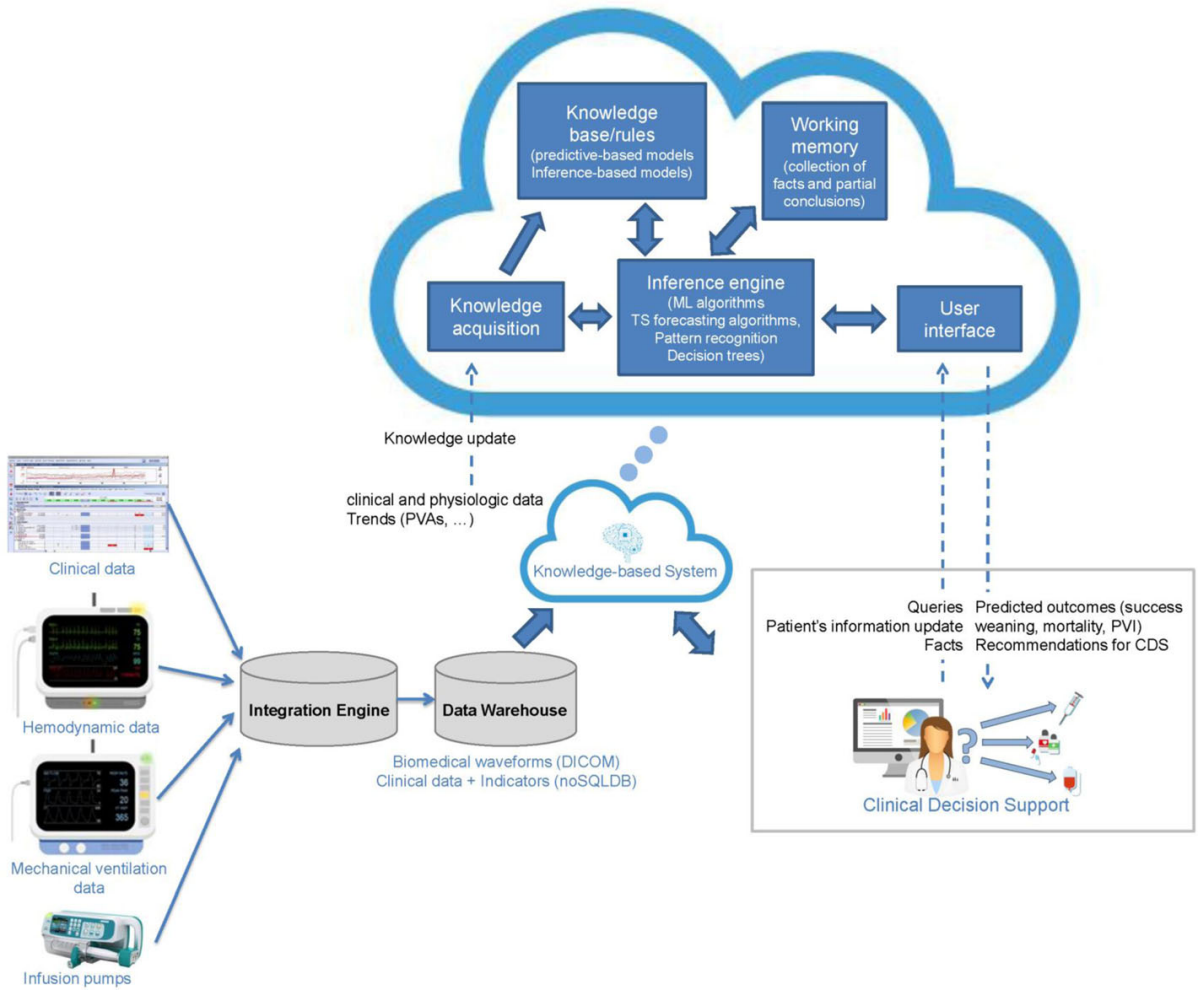
Future medical trends in real-time clinical decision making for mechanically ventilated critically patients in ICU. With adequate interoperability and data storage, clinical decision support systems based on big data analytics can automatically recognize patterns in data; moreover, these systems have the ability to improve continuously by “learning” from past and new inputs. Using the cloud for big data analytics makes it easier to make predictions and better understand trends

Fortunately, some steps are being taken in this direction. The Multiparameter and Intelligent Monitoring in Intensive Care (MIMIC) database contains thousands of ICU records reflecting daily clinical routines from a wide variety of sources, making it extremely useful for assessing clinical decision, monitoring algorithms, and testing new research hypothesis [64]. Another interesting initiative is the AEGLE project [65, 66], aimed at identifying ineffective efforts with big data analytics. AEGLE also addresses lung overstretching during assisted ventilation, identifying injurious high levels of pressure, and predicting the risk of this phenomenon developing within the next few minutes. A recent proof-of-concept study showed that it is feasible to use Hidden Markov Models to predict PVA in critically ill patients and to infer the probability that the number of asynchrony events will be above a given threshold [67]. All these approaches have potential health and economic benefits. Given the growing interest in devising better evidence-based care in the ICU, physicians should become familiar with the opportunities and challenges of big data [68] (Fig. 3).

## Conclusion

The results of observational studies evidence that poor patient-ventilator interaction might cause lung and vascular injury and thereby increase mortality. The effects of asynchronies on clinical outcomes remain to be clarified, but the type and presentation of asynchronies over time seems important. Together with damage resulting from the patient’s original disease, the short- and long-term consequences of poor patient-ventilator interaction can have devastating effects that hinder discharged patients’ complete return to normal activities. Therefore, critical care professionals must strive to improve patient-ventilator interaction. Observational studies could have some limitations on establishing association between patient-ventilator asynchronies and outcomes, and future multicenter studies with bigger population are needed. Finally, software solutions that can identify and analyze asynchronies online and offline may lead to better care and improve outcomes.

## Abbreviations

Acc: Overall accuracy
AI: Asynchrony index
CI: Confidence interval
EAdi: Electric activity diaphragm
ICC: Intraclass correlation coefficient
ICU: Intensive care unit
ML: Machine learning
NPV: Negative predictive value
OR: Odds ratio
PAW: Pressure airway
PPV: Positive predictive value
PuPV: Pulse pressure variation
PTSD: Post-traumatic stress disorder
PVA: Patient-ventilator asynchronies
ROC: Receiver operating characteristics
Se: Sensibility
SOFA: Sequential Organ Failure Assessment
Sp: Specificity
SVV: Stroke volume variation
*V*_t_: Tidal volume
